# Positive and Negative Affect More Concurrent among Blacks than Whites

**DOI:** 10.3390/bs7030048

**Published:** 2017-08-01

**Authors:** Maryam Moghani Lankarani, Shervin Assari

**Affiliations:** 1 Department of Psychiatry, University of Michigan, Ann Arbor 4250 Plymouth Road, SPC 5763, Ann Arbor, MI 48109-2700, USA; lankaranii@yahoo.com; 2 Medicine and Health Promotion Institute, Mollasadra Avenue, Tehran 1991, Iran; 3 Center for Research on Ethnicity, Culture and Health, School of Public Health, University of Michigan, Ann Arbor, MI 48109-2700, USA

**Keywords:** positive affect, negative affect, ethnic groups, Blacks, African Americans, Whites

## Abstract

Background: While positive and negative affect are inversely linked, people may experience and report both positive and negative emotions simultaneously. However, it is unknown if race alters the magnitude of the association between positive and negative affect. The current study compared Black and White Americans for the association between positive and negative affect. Methods: We used data from MIDUS (Midlife in the United States), a national study of Americans with an age range of 25 to 75. A total number of 7108 individuals were followed for 10 years from 1995 to 2004. Positive and negative affect was measured at baseline (1995) and follow-up (2004). Demographic (age and gender), socioeconomic (education and income) as well as health (self-rated health, chronic medical conditions, and body mass index) factors measured at baseline were covariates. A series of linear regressions were used to test the moderating effect of race on the reciprocal association between positive and negative affect at baseline and over time, net of covariates. Results: In the pooled sample, positive and negative affect showed inverse correlation at baseline and over time, net of covariates. Blacks and Whites differed in the magnitude of the association between positive and negative affect, with weaker inverse associations among Blacks compared to Whites, beyond all covariates. Conclusion: Weaker reciprocal association between positive and negative affect in Blacks compared to Whites has implications for cross-racial measurement of affect and mood, including depression. Depression screening programs should be aware that race alters the concordance between positive and negative affect domains and that Blacks endorse higher levels of positive affect compared to Whites in the presence of high negative affect.

## 1. Introduction

In 1969, Norman Bradburn showed that positive and negative affect are two separate, but inter-connected emotions with moderate negative associations [1,2]. Positive affect and emotions include happiness, joy, contentment, interest and love [3,4,5,6,7]; negative affect and emotions include sadness, guilt, fear, anger, and disgust [8,9,10]. While positive affect promotes health [11] and reduces risk of mortality [12] via better psychological adjustment [13], negative affect increases risk of chronic disease [14] as well as mortality [15] via vigilance, threat [16], and an unhealthy lifestyle [17].

Positive affect, a predictor of creation [18] and openness [19], is linked to lower autonomic reactivity [20] and better physical and mental health [21]. Constant high levels of negative affect, however, predisposes individuals to emotional and health problems [8,9,10]. The overall role of negative affect as a common risk factor for a wide range of emotional and psychiatric disorders, such as anxiety and depression, is well established, and may partially explain why several disorders tend to co-occur [22].

In addition to their physical health effects [23], positive and negative affect have major implications for diagnosis of mood disorders, particularly depression [24]. Positive and negative emotions compose major factors in mood disorders such as depression [25,26,27] and bipolar disorder [28]. As racial groups differ in their tendency to experience or express positive and negative affect [29,30], there is a need for cross-racial studies that compare racial groups for association between positive and negative affect [31,32,33,34,35,36,37,38].

Whether or not race alters simultaneous experience and presentation of positive and negative affect is still unknown [29,30]. While some studies have suggested that the factorial structure of depression scales is invariant across racial groups [39,40,41], other studies have shown that the degree by which positive and negative affect correlate may depend on race and ethnicity [29,42,43]. While there is an ongoing debate whether or not positive and negative affect similarly represent depression among Whites and Blacks [38,39,40,41,44,45,46,47,48,49,50], there are studies showing higher reliability of negative affect despite lower reliability of depression measures for Blacks compared to Whites [40,41,44]. Thus, positive and negative affect domains may differently compose the presentation of depression for Blacks and Whites [39].

In a recent study by Moazen-Zadeh and Assari, the item loading for the item “I was happy” to the Center for Epidemiologic Studies Depression Scale (CES-D) measure was 0.68 and −0.66 respectively for Blacks and Whites [29]. In another study, by Assari et al., negative affect showed stronger correlations with the Composite International Diagnostic Interview (CIDI)-based diagnosis of clinical depression in Blacks compared to Whites, while positive affect was similarly linked to MDD across race groups [45]. A national study found considerable variation in item loadings of the CES-D scale between Blacks and Whites. While negative affect items showed a better loading for Blacks, the positive affect items better loaded for Whites [5]. Canady et al. observed that the positive item “was happy” had different loadings on a depression scale between Blacks and Whites [43]. All these studies suggest that positive and negative affect may differently correlate between Blacks and Whites. These findings may help us better understand why correlates of depression and affect vary for Whites and Blacks [51,52,53], and why Blacks, who report higher levels of depressive symptoms (negative affect), do not endorse DSM criteria for the clinical disorder [29,45,54].

While positive and negative affect are main factors in several depression scales [41,49,50,55], different populations may differ in how they experience or express positive and negative affect simultaneously [41,55]. Considering the gap in the literature on racial and ethnic variation in the concordance between positive and negative affect endorsement [29,41,42,43,45,46,47,48], more research is needed on moderating effects of race and ethnicity on the reciprocal links between positive and negative affect [41,56,57].

We conducted the current study on racial differences in the bidirectional links between positive and negative affect, using a national sample of adults in the United States.

## 2. Methods

### 2.1. Design

This longitudinal study used data from MIDUS (http://midus.wisc.edu), a 10 year longitudinal study conducted between 1995 and 2004. The study was a national cohort study of over 7000 American adults (aged 25 to 74). The study was carried out by the MacArthur Midlife Research Network. The main purpose of the study was to understand the role of psychosocial factors in age related variation in physical and mental health over the life course [58,59,60,61,62].

### 2.2. Data Collection

The survey used a multimodal data collection strategy, which was composed of a computer-assisted personal interview (CAPI), a computer-assisted telephone interview (CATI), a mail questionnaire, a telephone interview, and a face-to-face interview. First, the study employed an initial 30-minute phone interview followed by a set (two) of self-administered questionnaires (SAQs). SAQs were mailed to individuals who completed the phone interview [58,59,60,61,62].

### 2.3. Ethics

The study protocol was approved by the University of Michigan Institutional Review Board (IRB), and written informed consent was obtained for all participants. The study was funded by the National Institute on Aging (NIA). Monetary incentives were given at both Wave 1 and Wave 2 for compensation (US $20 for completion of MIDUS 1 surveys and up to US $60 for completion of MIDUS 2 surveys).

### 2.4. Participants and Sampling

The study used random digit dialing (RDD), which is a method for selecting people for involvement in telephone statistical surveys by generating telephone numbers at random in order to enroll a random sample of adults. The national RDD survey used telephone numbers within the continental United States as the sampling frame. The study used an oversampling in five cities (related to geographic-specific agendas), resulting in a baseline RDD sample of 4244 individuals. The sibling sample was then generated by a random selection of 529 cases from the RDD sample that had at least one sibling. Limited to siblings within a family that had the same biological mother and father, the study collected data from 950 siblings. The study also enrolled a twin sample, which used a two-part sampling design. The first part involved screening a representative national sample of approximately 50,000 households for the presence of a twin (as part of ongoing national omnibus surveys). The second part involved contacting the twin households and attempting to recruit twins (also aged 25–74) to participate in the survey. Cooperating twins were asked to provide contact information for their co-twin. The twin sample was ultimately composed of 957 twin pairs (*n* = 1914) [58,59,60,61,62]. As 1187 individuals were neither Whites nor Blacks, this study only included 5921 White and Black individuals.

Data collection of wave 1 was conducted in 1995 and 1996. The follow-up data collection was conducted in 2004 and 2005. Advance letters with an accompanying brochure were sent to all Time-1 participants, to remind them about their past participation and to inform them that an interviewer will contact them for the initial telephone survey in near future. After a phone interview, which lasted 30 min on average, participants received two SAQs via mail [58,59,60,61,62].

### 2.5. Follow-Up Data

From the total 7108 participants who were enrolled at baseline (completing the phone survey at MIDUS 1), data were gathered for 4963 (70%) at MIDUS 2 9–10 years later. Overall retention rate was 75% in the MIDUS (adjusted for mortality). Major causes for non-participation at MIDUS 2 included refusal (12%), could not be contacted (10%), too ill to be interviewed (8%), or deceased [58,59,60,61,62].

### 2.6. Measures

Predictor variables were those prominent in published research on survey participation and retention, including 7 core demographic variables and 10 physical health variables from either the baseline telephone interview or the SAQ.

### 2.7. Demographic Characteristics

Demographic variables were collected at baseline in 1995 and included age (continuous), gender (0 = male (reference group), 1 = female), and race (0 = Whites (reference group), 1 = Blacks).

*Socioeconomic status.* Socio-economic variables included educational level (1 = less than high school, 2 = high school graduate or equivalent, 3 = some college, 4 = college graduate or more) and personal income. Both variables were operationalized as continuous measures.

*Physical Health.* The following physical health variables were included in the study: self-rated health (SRH) (1 = worst, 10 = best), number of chronic medical conditions (CMC), and body mass index (BMI). All measures were treated as continuous measures. While a higher score indicated better SRH, higher scores for CMC and BMI reflected worse health.

*Positive affect.* Using a scale developed by Mroczek and Kolarz (1998), [63] positive affect was assessed using six items referring to the question: “During the past 30 days, how much of the time did you feel…” Items included “cheerful”, “in good spirits”, “extremely happy”, “calm and peaceful”, “satisfied”, and “full of life”. Responses ranged from 1 (all of the time) to 5 (none of the time) for each item [63]. Mean positive affect scores were computed if at least one of the affect items were completed. Greater scores reflected more positive affect, with possible scores ranging from 1 to 5. Internal consistency (reliability) was very good (α = 0.91 for all, 0.91 for Whites, 0.92 for Blacks). Other studies have used this scale [64,65,66].

*Negative affect.* Using a scale developed by Mroczek and Kolarz (1998), [63] negative affect was assessed in response to six items referring to the question: “During the past 30 days, how much of the time did you feel…” Negative affect items included “so sad”, “nervous”, “restless or fidgety”, “hopeless”, “worthless”, and “everything was an effort”. Responses ranged from 1 (all of the time) to 5 (none of the time) [63]. Mean negative affect scores were computed if at least one of the affect items were completed. Greater scores reflected more negative affect, with possible scores ranging from 1 to 5. Internal consistency (reliability) was excellent (α = 0.86 for all, 0.86 for Whites, 0.87 for Blacks). Other studies have used this scale [64,65,66].

### 2.8. Statistical Note

We used SPSS 20.0 for Windows (IBM Inc. Armonk, NY, USA) for data analysis. For univariate analysis we reported frequencies, percentages, and mean (standard deviations) in the pooled sample, as well as based on race. For bivariate associations, we used independent sample t test, chi square test, as well as Pearson correlation test. For multivariable analysis, we ran a series of linear regressions in the pooled sample, with positive and negative affect as independent and dependent variables, respectively. In our first models, only demographics and socio-economics were controlled for. Subsequently, we controlled for health factors. First, we ran models without the interaction term. Then, we added the race by affect interaction term. We ran models with wave 1 and wave 2 positive and negative affect as outcomes. In the next step, we ran models stratified based on race. Unstandardized regression coefficient (b), standard error (SE), 95% Confidence Intervals (CI), and *p* values were reported.

## 3. Results

Table 1 presents the results of descriptive analysis in the pooled sample and also based on race. Compared to Whites, Blacks were younger, more frequently women, and had lower education and income. Blacks had worse CMC, SRH and BMI compared to Whites. Blacks also had higher positive affect at baseline compared to Whites. Blacks and Whites were similar in negative affect at baseline.

Table 2 presents the correlation matrix of the study variables in Whites and Blacks. While positive affect in wave 1 and 2 were correlated in both racial groups, the magnitude of the correlation between positive and negative affect were stronger for Whites than Blacks. Age was correlated with wave 1 positive and negative affect in Whites but not Blacks. Education and income also showed better correlations with wave 1 and wave 2 positive and negative affect in Whites than Blacks.

Table 3a summarizes the results of four linear regression models to test the effects of negative affect in wave 1 on positive affect in wave 1. Model 1-a only included demographic and socioeconomic factors without any interaction term. Model 2-a included demographic and socioeconomic factors, however also added the race by negative affect in wave 1 interaction term. Model 3-a included health factors as controls with no interaction term. Model 4-a included health factors as controls, with race by negative affect in wave 1 interaction term. According to our models, baseline negative affect showed strong inverse correlation with wave 1 positive affect, net of socioeconomic (Model 1-a) and health status (Model 3-a). Significant difference was found between Blacks and Whites for the association between positive and negative affect such that the inverse association was weaker for Blacks compared to Whites in a model with demographic and socioeconomic (Model 2-a) as well as health status (Model 4-a) (Table 3a).

Table 3b provides a summary of four linear regression models with wave 1 positive affect as the independent variable and wave 1 negative affect as the dependent variable. Baseline positive affect showed strong inverse correlation with wave 1 negative affect, net of socioeconomic (Model 1-b) and health status (Model 3-b). Significant difference was found between Blacks and Whites for the association between positive and negative affect such that the inverse association was weaker for Blacks compared to Whites in the presence of demographic and socioeconomic (Model 2-b) as well as health status (Model 4-b) (Table 3b).

Table 4a presents the results of four linear regression models with wave 1 negative affect as the predictor and wave 2 positive affect as the outcome. According to our models, baseline negative affect showed strong inverse correlation with wave 1 positive affect, net of demographic and socioeconomic (Model 1-a) as well as health status (Model 3-a). Significant difference was found between Blacks and Whites for the association between positive and negative affect such that the inverse association was weaker for Blacks compared to Whites net of demographic and socioeconomic (Model 2-a) as well as health status (Model 4-a) (Table 4a).

Table 4b shows the results of four linear regression models with wave 2 negative affect as the outcome. According to our models, baseline positive affect showed inverse correlation with wave 2 negative affect, net of demographic and socioeconomic (Model 1-b) and health status (Model 3-b). Significant difference was found between Blacks and Whites for the association between positive and negative affect such that the inverse association was weaker for Blacks compared to Whites net of demographic and socioeconomic (Model 2-b) as well as health status (Model 4-b) (Table 4b).

Table 5 summarizes predictors of wave 1 (Table 5a) positive and (Table 5a) negative affect in Whites and Blacks. Based on this table, net of demographic and socioeconomic as well as health status, positive affect showed inverse correlation with negative affect among Whites and Blacks. Regression coefficients were larger for Whites than Blacks.

Table 6 presents predictors of wave 2 positive (Table 6a) and negative (Table 6b) affect in Whites and Blacks. Based on this table, net of demographic and socioeconomic as well as health status, wave 1 positive affect showed inverse correlation with wave 2 negative affect among Whites but not Blacks. Wave 1 negative affect also showed inverse correlation with wave 2 positive affect among Whites but not Blacks.

## 4. Discussion

In our study, Black and White Americans differed in the magnitude of the inverse correlation between positive and negative affect at baseline and over time. According to our study, the negative association between positive and negative affect was weaker for Black compared to White Americans. These differential correlations remained significant at baseline and over time, net of demographic, socioeconomic, and health status.

Our finding of a stronger link between positive and negative affect among White compared to Black Americans is consistent with previous findings showing weaker negative association between depressive symptoms and hopefulness among Whites than Blacks [67]. In the presence of similar depressive symptoms, Blacks maintain higher levels of hope than Whites, [67] and Blacks who endorse high negative affect maintain high positive affect as well. Black-White differences also exist in the magnitude of correlation between depression and evaluation of self [68]. Negative affect and depressive symptoms differently negative feelings and cognitions about self in Whites and Blacks [68]. The weaker effects of depression and depressive symptoms on physical health outcomes, such as incident chronic disease [69] and all-cause [70] and disease specific chronic disease [71], in Whites than Blacks are also known. In one study, depressive symptoms predicted future clinical depression in Whites but not Blacks [30], and in another study, race altered how depressive symptoms map on clinical depression [46].

Canady et al. found the item “I was happy” as the only item with different loadings between Blacks and Whites after applying the cross-group constraints [43]. In a recent study, Assari and Moazen-Zadeh found differences for several positive affect items, including the item “I was happy,” on a depression scale for Blacks and Whites. The study showed worse item loadings for positive items, namely “as good” and “hopeful,” in Blacks than Whites. In the final model with a very good fit to the data, the item “as good” showed poor loading for Whites and Blacks, however, the item “hopeful” showed good loading for Whites but poor loading for Blacks [42].

A recent body of evidence has shown major racial differences in socioeconomic and health correlates of negative emotions [51,52,53]. Negative emotions better predict medical conditions, obesity, and mortality in Whites than Blacks [52,53]. Based on Black-White health paradox, defined as less frequent depression despite a higher prevalence of chronic medical conditions among Blacks compared to Whites [54,72,73,74,75,76,77,78,79,80,81,82], adversities and negative emotions are more common among Blacks [69]. Our findings may explain why emotional disorders are not as common as expected in Blacks.

According to the “undoing hypothesis,” positive emotions are able to undo the harmful effects of negative emotions [5,7]. Maintaining higher levels of positive affect in the presence of negative affect in Blacks compared to Whites may explain the weaker effects of negative emotions on physical health of Blacks. In this view, positive affect operates as a buffer against harms associated with negative affect [7,20]. This phenomenon can explain why depressive symptoms predict incident chronic disease [69] and mortality [52,53] for Whites but not Blacks. Inflammation also better correlates with depression for Blacks than Whites [81,82]. This finding can also explain why depressive symptoms predict subsequent MDD among Whites but not Blacks [29]. This phenomenon may also explain why Blacks report better well-being (positive affect) than Whites, despite higher levels of stress, psychological distress, and depressive symptoms [83].

Our findings are in support of the literature that suggests positive and negative affect are separate but interconnected components of depression measurement, among both Blacks and Whites [25,26,27,44,84]. While this study investigated positive and negative emotions, there is a need to explore racial differences in correlations between emotions and other domains of depression such as somatic complaints and interpersonal problems [85,86,87,88]. Future research may also test if resources and assets such as self-esteem, social support, religion, or culture explain Black-White variation in the link between positive and negative affect.

Our findings on Black-White differences in the link between positive and negative affect have major implications for measurement of depression and depressive symptoms in ethnically diverse populations. Clinicians and researchers who work with racially diverse populations should be aware of race-specific links between positive and negative emotions. Compared to their White counterparts, Blacks with depression may endorse higher levels of positive affect, which may reduce chance of diagnosis of depression. We argue that positive and negative items of depression should not be simply summed among diverse populations. Instead, positive and negative domains should be considered as inter-connected but separate domains across racial groups. Domain-specific evaluation of mood may be a better option than calculating a sum score of positive and negative items. Considering such cross-ethnic variations may help with more accurate diagnosis and treatment of depression among ethnically diverse populations [85,89].

Our findings also have implications for epidemiological studies of depression across various racial groups. The differential link between positive and negative affect based on race causes measurement bias for cross-racial measurement of mood outcomes, including but not limited to depression. Programs that screen ethnically diverse samples for depression or depressive symptomatology should be aware of how race alters the concordance of positive and negative affect. There is still a need for further research on equivalence of depression, affect, and mood measurement among diverse racial and ethnic groups [51,90,91]. Future research should focus on cross-racial validation of measures of depression.

Our study is subject to at least three limitations. The major limitation of this study was not measuring culture [92,93]. Second, we did not include clinical diagnosis of MDD according to the DSM criteria (composite international diagnostic interview criteria were used). Third, we did not consider race by gender differences in this study. Despite how the race by gender intersection may shape experience and expression of positive and negative emotions [94], limited sample size of Blacks did not allow us to break our sample of Blacks to additional sub-groups. Despite this limitation, a unique strength of our study was using nationally representative data of American adults that resulted in nationally generalizable findings to the U.S. population.

In conclusion, our findings suggest that positive and negative affect have a weaker negative association in Blacks than Whites, suggesting that in the presence of similar negative affect, Blacks maintain higher levels of positive affect than Whites. These findings have health implications given the protective effect of positive affect (the “undoing hypothesis”). It is unknown whether racial differences in the magnitude of the correlation between positive and negative affect explains previously observed Black-White differences in psychosocial and medical correlates of depression. In addition, these findings are also important for measurement of depression. This finding calls into question the measurement equivalence of affect, mood, and depression outcomes among Blacks and Whites.

## 5. Conclusions

In summary, positive and negative affect are more concurrent in Blacks compared to Whites. This finding has implications for cross-racial measurement of affect and mood, including depression. This information may have implications for diagnosis and screening of depression in racially diverse populations.

## Figures and Tables

**Table 1:** Descriptive statistics in the pooled sample and based on race.

		All		Whites		Blacks	*p*
	**N**	**%**	**N**	**%**	**N**	**%**	
**Demographics**							
Gender							
Men	3395	47.8	2683	47.9	121	37.7	<0.001
Women	3632	51.1	2917	52.1	200	62.3	
	**Mean**	**SD**	**Mean**	**SD**	**Mean**	**SD**	
Age	46.38	13.00	47.30	12.92	44.42	12.54	<0.001
**Socioeconomics**							
Education	6.77	2.49	6.90	2.47	6.22	2.47	<0.001
Income	26,773.24	26,891.19	27,326.10	27,509.71	20,762.54	19,730.37	<0.001
Self-Rated Health	7.45	1.62	7.46	1.60	7.65	1.84	0.043
Chronic Medical Conditions	2.41	2.51	2.39	2.46	2.53	2.96	0.422
Body Mass Index	26.67	5.29	26.55	5.17	28.69	6.38	<0.001
**Outcomes**							
Negative Affect, Wave 1	1.53	0.62	1.53	0.61	1.53	0.65	0.879
Negative Affect, Wave 2	1.51	0.58	1.50	0.56	1.67	0.83	0.001
Positive Affect, Wave 1	3.39	0.73	3.38	0.72	3.52	0.77	0.002
Positive Affect, Wave 2	3.43	0.71	3.42	0.70	3.55	0.79	0.073

**Table 2:** Correlation matrix of the study variables in White and Black Americans.

	1	2	3	4	5	6	7	8	9	10	11
1 Gender (Women)	1	0.01	−0.10 **	−0.36 **	0.00	0.12 **	−0.10 **	0.08 **	0.08 **	−0.03 *	−0.02
2 Age	−0.12 *	1	−0.11 **	−0.15 **	−0.03 *	0.18 **	0.11 **	−0.10 **	−0.10 **	0.10 **	0.14 **
3 Education	−0.03	−0.10	1	0.36 **	0.10 **	−0.13 **	−0.10 **	−0.09 **	−0.13 **	0.02	0.04 *
4 Income	−0.26 **	−0.04	0.36 **	1	0.10 **	−0.16 **	0.00	−0.10 **	−0.14 **	0.04 **	0.07 **
5 Self-Rated Health	−0.07	−0.02	0.02	0.17 **	1	−0.42 **	−0.24 **	−0.36 **	−0.28 **	0.39 **	0.31 **
6 Chronic Medical Conditions	0.13 *	0.13*	−0.10	−0.20 **	−0.33 **	1	0.17 **	0.41 **	0.30 **	−0.33 **	−0.24 **
7 Body Mass Index	0.14 *	0.09	−0.08	−0.09	−0.25 **	0.15 **	1	0.06 **	0.06 **	−0.05 **	−0.06 **
8 Negative Affect, Wave 1	0.15 **	−0.05	−0.05	−0.15 **	−0.21 **	0.27 **	0.10	1	0.51 **	−0.64 **	−0.39 **
9 Negative Affect, Wave 2	0.12	−0.21 *	−0.15	−0.19 *	−0.23 **	0.04	0.20 *	0.40 **	1	−0.39 **	−0.62 **
10 Positive Affect, Wave 1	−0.16 **	0.06	−0.05	0.03	0.30 **	−0.18 **	−0.05	−0.46 **	−0.21 *	1	0.53 **
11 Positive Affect, Wave 2	−0.04	0.16*	−0.05	0.03	0.44 **	−0.04	−0.13	−0.28 **	−0.44 **	0.51 **	1

Whites, up diagonal; Blacks, low diagonal.* *p* < 0.05; ** *p* < 0.01.

**Table 3:** Predictors of wave 1 positive and negative affect in the pooled sample.

	B(SE)	95% CI	*P*	B(SE)	95% CI	*P*	B(SE)	95% CI	*P*	B(SE)	95% CI	*P*
(**a**)												
**Outcome:** Wave 1 Positive Affect												
		*Model 1-a*			*Model 2-a*			*Model 3-a*			*Model 4-a*	
Race (Blacks)	0.14 (0.03)	0.07–0.20	<0.001	−2.34 (0.15)	−2.64–−2.06	<0.001	0.12 (0.03)	0.06–0.19	<0.001	−2.23 (0.07)	−2.52–−1.94	<0.001
Age	0.00 (0.00)	0.00–0.00	0.002	0.00 (0.00)	0.00–0.00	0.001	0.00 (0.00)	0.00–0.00	<0.001	0.00 (0.00)	0.00–0.00	<0.001
Gender	0.02 (0.02)	−0.01–0.05	0.242	0.03 (0.02)	0.00–0.06	0.070	0.01 (0.02)	−0.02–0.04	0.489	0.02 (0.01)	−0.01–0.05	0.198
Education	−0.01 (0.00)	−0.01–0.00	0.056	−0.00 (0.00)	−0.01–0.00	0.096	−0.01 (0.00)	−0.01–0.00	0.005	−0.01 (0.00)	−0.01–0.00	0.011
Income	−0.00 (0.00)	0.00–0.00	0.321	−0.00 (0.00)	0.00–0.00	0.435	−0.00 (0.00)	0.00–0.00	0.080	−0.00 (0.00)	0.00–0.00	0.125
Self-Rated Health							0.08 (0.01)	0.07–0.09	<0.001	0.08 (0.00)	0.07–0.09	<0.001
Chronic Medical Conditions							−0.01 (0.00)	−0.020–−0.00	0.002	−0.01 (0.00)	−0.02–−0.00	0.002
Body Mass Index							−0.00 (0.00)	−0.00–0.01	0.195	−0.00 (0.00)	−0.00–0.01	0.147
Negative Affect, Wave 1	−0.76 (0.01)	−0.78–−0.74	<0.001	−0.74 (0.01)	−0.76–−0.71	<0.001	−0.67 (0.01)	−0.70–−0.64	<0.001	−0.65 (0.01)	−0.68–−0.62	<0.001
Negative Affect × Black				0.70 (0.04)	0.62–0.78	<0.001				0.67 (0.04)	0.59–0.75	<0.001
(**b**)												
**Outcome:** Wave 1 Negative Affect												
		*Model 1-b*			*Model 2-b*			*Model 3-b*			*Model 4-b*	
Race (Blacks)	0.04 (0.03)	−0.01–0.10	0.136	−1.02 (0.07)	−1.15–−0.89	<0.001	0.04 (0.03)	−0.01–0.10	0.144	−0.95 (0.07)	−1.08–−0.82	<0.001
Age	0.00 (0.00)	0.00–0.00	<0.001	−0.00 (0.00)	0.00–0.00	<0.001	0.00 (0.00)	−0.01–0.00	<0.001	0.00 (0.00)	−0.01–0.00	<0.001
Gender	0.04 (0.01)	0.01–0.07	0.003	0.04 (0.01)	0.01–0.06	0.006	0.02 (0.01)	−0.01–0.04	0.156	0.01 (0.01)	−0.01–0.04	0.243
Education	−0.01 (0.00)	−0.02–−0.01	<0.001	−0.01 (0.00)	−0.02–−0.01	<0.001	−0.01 (0.00)	−0.01–0.00	<0.001	−0.01 (0.00)	−0.01–0.00	<0.001
Income	0.00 (0.00)	0.00–0.00	<0.001	0.00 (0.00)	0.00–0.00	<0.001	0.00 (0.00)	0.00–0.00	0.005	0.00 (0.00)	0.00–0.00	0.006
Self-Rated Health							−0.02 (0.00)	−0.03–−0.01	<0.001	−0.02 (0.00)	−0.03–−0.01	<0.001
Chronic Medical Conditions							0.05 (0.00)	0.05–0.06	<0.001	0.05 (0.00)	0.05–0.06	<0.001
Body Mass Index							0.00 (0.00)	0.00–0.00	0.108	0.00 (0.00)	0.00–0.00	0.057
Positive Affect, Wave 1	−0.54 (0.01)	−0.55–−0.52	<0.001	−0.52 (0.01)	−0.54–−0.50	<0.001	−0.45 (0.01)	−0.47–−0.43	<0.001	−0.44 (0.01)	−0.46–−0.42	<0.001
Positive Affect × Black				0.70 (0.04)	0.62–0.78	<0.001				0.65 (0.04)	0.57–0.73	<0.001

**Table 4:** Predictors of wave 2 positive and negative affect in the pooled sample.

	b(SE)	95% CI for b	*P*	b(SE)	95% CI for b	*P*	b(SE)	95% CI for b	*P*	b(SE)	95% CI for b	*P*
(**a**)												
**Outcome:** Wave 2 Positive Affect												
		*Model 1-a*			*Model 2-a*			*Model 3-a*			*Model 4-a*	
Race (Blacks)	0.16 (0.06)	0.05−0.27	0.005	−0.96 (0.27)	−1.48–−0.43	<0.001	0.10 (0.06)	−0.01−0.21	0.072	−0.87 (0.26)	−10.39–−0.35	0.001
Age	0.01 (0.00)	0.00−0.01	<0.001	0.01 (0.00)	0.00−0.01	<0.001	0.01 (0.00)	0.00−0.01	<0.001	−0.01 (0.00)	0.00−0.01	<0.001
Gender (Female)	0.05 (0.02)	0.01−00.10	0.027	0.05 (0.02)	0.01−0.10	0.019	0.04 (0.02)	0.00−0.09	0.054	0.05 (0.02)	0.00−0.09	0.039
Education	0.00 (0.00)	−0.01−0.01	0.594	0.00 (0.00)	−0.01−0.01	0.590	0.00 (0.00)	−0.01−0.01	0.854	0.00 (0.00)	−00.01−00.01	0.866
Income ($)	0.00 (0.00)	0.00−00.00	0.003	0.00 (0.00)	0.00−0.00	0.003	0.00 (0.00)	0.00−0.00	0.018	0.00 (0.00)	0.00−0.00	0.014
Self-Rated Health (1–10)							0.09 (0.01)	0.08−0.11	<0.001	0.09 (00.01)	0.08−0.11	<0.001
Chronic Medical Conditions (n)							0.02 (0.01)	−0.03–−0.01	0.002	−00.02 (0.01)	−0.03–−0.01	0.002
Body Mass Index (lb/in2)							0.00 (0.00)	0.00−0.01	0.679	0.00 (0.00)	0.00−0.01	0.668
Negative Affect, Wave 1	−00.45 (0.02)	−0.49–−0.42	<0.001	−00.45 (0.02)	−0.48–−0.41	<0.001	−0.35 (0.02)	−0.39–−0.31	<0.001	−00.35 (0.02)	−0.39–−0.31	<0.001
Negative Affect × Black				0.31 (0.07)	0.17−0.46	<0.001				0.27 (0.07)	0.13−0.42	<0.001
(**b**)												
**Outcome:** Wave 2 Negative Affect												
		*Model 1-b*			*Model 2-b*			*Model 3-b*			*Model 4-b*	
Race (Blacks)	0.17 (0.05)	0.08−00.26	<0.001	−00.26 (0.10)	−0.46–−0.05	0.013	0.17 (0.05)	0.08−0.26	<0.001	−00.23 (0.10)	−0.43–−0.03	0.024
Age	0.00 (0.00)	−0.01−0.00	<0.001	0.00 (0.00)	−0.01−0.00	<0.001	−0.01 (0.00)	−0.01−0.00	<0.001	−00.01 (0.00)	−0.01−0.00	<0.001
Gender (Female)	0.02 (0.02)	−0.01−0.06	0.205	0.02 (0.02)	−0.01−0.06	0.242	0.01 (0.02)	−0.03−0.04	0.758	0.00 (0.02)	−0.03−0.04	0.824
Education	−00.02 (0.00)	−0.03–−0.01	<0.001	−00.02 (0.00)	−0.03–−0.01	<0.001	−00.02 (0.00)	−0.03–−0.01	<0.001	−00.02 (0.00)	−0.03–−0.01	<0.001
Income ($)	0.00 (0.00)	0.00−00.00	<0.001	0.00 (0.00)	0.00−0.00	<0.001	0.00 (0.00)	0.00−0.00	<0.001	0.00 (0.00)	0.00−0.00	<0.001
Self-Rated Health (1–10)							−0.03 (0.01)	−0.05–−0.02	<0.001	−0.03 (0.01)	−0.04–−0.02	<0.001
Chronic Medical Conditions (n)							0.04 (0.00)	0.03−0.05	<0.001	0.04 (0.00)	0.03−0.05	<0.001
Body Mass Index (lb/in2)							0.00 (0.00)	0.00−0.00	0.665	0.01 (0.00)	0.00−0.00	0.654
Positive Affect, Wave 1	−0.30 (0.01)	−0.32–−0.27	<0.001	−0.29 (0.01)	−0.31–−0.27	<0.001	−0.23 (0.01)	−0.26–−0.20	<0.001	−0.23 (0.01)	−0.25–−0.20	<0.001
Positive Affect × Black				0.28 (0.06)	0.16−0.40	<0.001				0.26 (0.06)	0.15−0.38	<0.001

**Table 5:** Predictors of wave 1 positive and negative affect in White and Black Americans.

	B(SE)	95% CI	*P*	B(SE)	95% CI	*P*	B(SE)	95% CI	*P*	B(SE)	95% CI	*P*
				Whites						Blacks		
(**a**)												
**Outcome:** Wave 1 Positive Affect												
		*Model 1*			*Model 2*			*Model 3*			*Model 4*	
Age	0.00 (0.00)	0.00–0.00	0.002	0.00 (0.00)	0.00–0.00	<0.001	0.00 (0.00)	-0.01–0.01	0.802	0.00 (0.00)	0.00–0.01	0.447
Gender (Female)	0.03 (0.02)	0.00–0.06	0.058	0.02 (0.02)	−0.01–0.05	0.198	−0.22 (0.09)	−0.38–−0.05	0.013	−0.18 (0.09)	−0.36–−0.01	0.041
Education	−0.01 (.00)	0–0.01–0.00	0.069	0–0.01 (.00)	−0.02–0.00	0.006	−0.01 (0.02)	−0.05–0.02	0.467	−0.01 (0.02)	−0.04–0.03	0.642
Income (USD1000)	0.00 (0.00)	0.00–0.00	0.505	0.00 (0.00)	0.00–0.00	0.178	0.00 (0.00)	0.00–0.00	0.232	0.00 (0.00)	0.00–0.00	0.086
Self-Rated Health (1–10)				0.08 (0.01)	0.07–0.09	<0.001				0.08 (0.02)	0.04–0.13	0.001
Chronic Medical Conditions (n)				−0.01 (0.00)	−0.02–0.00	0.004				−0.01 (0.02)	−0.04–0.02	0.352
Body Mass Index (lb/in2)				0.00 (0.00)	0.00–0.00	0.210				0.00 (0.01)	−0.01–0.02	0.666
Negative Affect, Wave 1	−0.77 (0.01)	−0.80–−0.75	0.000	−0.68 (0.01)	−0.71–−0.65	<0.001	−0.55 (0.06)	−0.67–−0.43	<0.001	−0.49 (0.07)	−0.62–−0.36	<0.001
(**b**)												
**Outcome**: Wave 1 Negative Affect												
		*Model 1*			*Model 2*			*Model 3*			*Model 4*	
Age	0.00 (0.00)	0.00–0.00	<0.001	0.00 (0.00)	−0.01–0.00	<0.001	0.00 (0.00)	−0.01–0.00	0.467	0.00 (0.00)	−0.01–0.00	0.219
Gender (Female)	0.04 (0.01)	0.02–0.07	0.001	0.02 (0.01)	0.00–0.05	0.113	0.01 (0.07)	−0.13–0.16	0.841	−0.01 (0.08)	−0.16–0.014	0.923
Education	−0.01 (0.00)	−0.02–−0.01	<0.001	−0.01 (0.00)	−0.01–0.00	<0.001	0.00 (0.01)	−0.03–0.03	0.831	−0.01 (0.01)	−0.03–0.02	0.709
Income ($)	0.00 (0.00)	0.00–0.00	<0.001	0.00 (0.00)	0.00–0.00	0.012	0.00 (0.00)	0.00–0.00	0.018	0.00 (0.00)	0.00–0.00	0.114
Self-Rated Health (1–10)				−0.02 (0.00)	−0.03–−0.01	<0.001				−0.02 (0.02)	−0.06–0.02	0.378
Chronic Medical Conditions (n)				0.05 (0.00)	0.05–0.06	<0.001				0.04 (0.01)	0.02–0.07	0.001
Body Mass Index (lb/in2)				0.00 (0.00)	0.00–0.00	0.059				0.00 (0.01)	−0.01–0.01	0.560
Positive Affect, Wave 1	−0.55 (0.01)	−0.56–−0.53	<0.001	−0.46 (0.01)	−0.48–−0.44	<0.001	−0.40 (0.04)	−0.48–−0.31	<0.001	−0.35 (0.05)	−0.44–−0.26	<0.001

**Table 6:** Predictors of wave 1 positive and negative affect in White and Black Americans.

	B(SE)	95% CI	*P*	B(SE)	95% CI	*P*	B(SE)	95% CI	*P*	B(SE)	95% CI	*P*
(**a**)												
**Outcome:** Wave 2 Positive Affect												
		*Model 1*			*Model 2*			*Model 3*			*Model 4*	
Age	0.01 (0.00)	0.00–0.01	<0.001	0.01 (0.00)	0.00–0.01	<0.001	0.00 (0.01)	−0.01–0.01	0.381	0.00 (0.01)	−0.01–0.01	0.683
Gender (Female)	0.04 (0.02)	0.00–0.08	0.075	0.04 (0.02)	−0.01–0.08	0.100	0.13 (0.14)	−0.14–0.41	0.335	0.06 (0.13)	−0.20–0.33	0.628
Education	0.01 (0.00)	0.00–0.01	0.224	0.00 (.00)	−0.01–0.01	0.546	−0.02 (0.02)	−0.07–0.03	0.351	−0.02 (0.02)	−0.06–0.03	0.492
Income (USD1000)	0.00 (0.00)	0.00–0.00	<0.001	0.00 (.00)	0.00–0.00	0.001	0.00 (0.00)	0.00–0.00	0.583	0.00 (0.00)	0.00–0.00	0.655
Self-Rated Health (1–10)				0.05 (0.01)	0.03–0.06	<0.001				0.15 (0.04)	0.08–0.23	0.000
Chronic Medical Conditions (n)				−0.02 (0.01)	−0.03–−0.01	0.002				0.06 (0.02)	0.01–0.11	0.015
Body Mass Index (lb/in2)				0.00 (0.00)	0.00–0.00	0.911				−0.01 (0.01)	−0.03–0.01	0.333
Positive Affect, Wave 1	0.46 (0.02)	0.43–0.50	<0.001	0.43 (0.02)	0.39–0.47	<0.001	0.51 (0.09)	0.33–0.69	<0.001	0.43 (0.09)	0.26–0.61	<0.001
Negative Affect, Wave 1	−0.09 (0.02)	−0.14–−0.05	<0.001	−0.06 (0.02)	−0.10–−0.01	0.020	−0.05 (0.09)	−0.23–0.13	0.578	−0.06 (0.09)	−0.23–0.12	0.504
(**b**)												
**Outcome:** Wave 2 Negative Affect												
		*Model 1*			*Model 2*			*Model 3*			*Model 4*	
Age	0.00 (0.00)	0.00–0.00	<0.001	0.00 (0.00)	0.00–0.00	<0.001	−0.01 (0.01)	−0.02–0.00	0.044	−0.01 (0.01)	−0.02–0.00	0.049
Gender (Female)	0.01 (0.02)	−0.03–0.04	0.639	0.00 (0.02)	−0.04–0.03	0.900	0.05 (0.15)	−0.26–0.35	0.764	0.02 (0.16)	−0.29–0.34	0.876
Education	−0.02 (0.00)	−0.03–−0.01	<0.001	−0.02 (0.00)	−0.02–−0.01	<0.001	−0.03 (0.03)	−0.08–0.02	0.266	−0.03 (0.03)	−0.08–0.03	0.310
Income ($)	0.00 (0.00)	0.00–0.00	<0.001	0.00 (0.00)	0.00–0.00	<0.001	0.00 (0.00)	0.00–0.00	0.395	0.00 (0.00)	0.00–0.00	0.368
Self-Rated Health (1–10)				−0.02 (0.01)	−0.04–−0.01	<0.001				−0.04 (0.05)	−0.13–0.06	0.433
Chronic Medical Conditions (n)				0.02 (0.00)	0.01–0.03	<0.001				−0.03 (0.03)	−0.08–0.03	0.348
Body Mass Index (lb/in2)				0.00 (0.00)	0.00–0.00	0.915				0.02 (0.01)	0.00–0.04	0.057
Negative Affect, Wave 1	0.41 (0.02)	0.37–0.45	<0.001	0.36 (0.02)	0.32–0.40	<0.001	0.36 (0.10)	0.16–0.56	0.001	0.37 (0.11)	0.17–0.58	0.001
Positive Affect, Wave 1	−0.08 (0.02)	−0.11–−0.05	<0.001	−0.06 (0.02)	−0.10–−0.03	<0.001	−0.03 (0.10)	−0.23–0.17	0.775	−0.02 (0.11)	−0.24–0.20	0.845
